# BAY 11-7082 Is a Broad-Spectrum Inhibitor with Anti-Inflammatory Activity against Multiple Targets

**DOI:** 10.1155/2012/416036

**Published:** 2012-06-15

**Authors:** Jaehwi Lee, Man Hee Rhee, Eunji Kim, Jae Youl Cho

**Affiliations:** ^1^College of Pharmacy, Chung-Ang University, Seoul 156-756, Republic of Korea; ^2^Laboratory of Physiology & Cell Signaling, College of Veterinary Medicine, Kyungpook National University, Daegu 702-701, Republic of Korea; ^3^Department of Genetic Engineering, Sungkyunkwan University, Suwon 440-746, Republic of Korea

## Abstract

BAY 11-7082 (BAY) is an inhibitor of **κ**B kinase (IKK) that has pharmacological activities that include anticancer, neuroprotective, and anti-inflammatory effects. In this study, BAY-pharmacological target pathways were further characterized to determine how this compound simultaneously suppresses various responses. Primary and cancerous (RAW264.7 cells) macrophages were activated by lipopolysaccharide, a ligand of toll-like receptor 4. As reported previously, BAY strongly suppressed the production of nitric oxide, prostaglandin E_2_, and tumor necrosis factor-**α** and reduced the translocation of p65, major subunit of nuclear factor-**κ**B, and its upstream signaling events such as phosphorylation of I**κ**B**α**, IKK, and Akt. In addition, BAY also suppressed the translocation and activation of activator protein-1, interferon regulatory factor-3, and signal transducer and activator of transcription-1 by inhibiting the phosphorylation or activation of extracellular signal-related kinase, p38, TANK-binding protein, and Janus kinase-2. These data strongly suggest that BAY is an inhibitor with multiple targets and could serve as a lead compound in developing strong anti-inflammatory drugs with multiple targets in inflammatory responses.

## 1. Introduction

Inflammatory signals activate inflammatory cells such as blood vessel epithelial cells, macrophages, neutrophils, mast cells, and lymphocytes to produce inflammatory mediators These include cytokines such as interleukin (IL)-1, IL-6, and tumor necrosis factor (TNF)-*α*; chemokines; toxic molecules including nitric oxide (NO); lipidic mediators including prostaglandin E_2_ (PGE_2_) [1, 2]. For these events, new transcriptional and translational processes are required for signaling cascades that are generated by the interaction between a receptor (e.g., the toll-like receptor 4 [TLR4]) and a ligand lipopolysaccharide (LPS) [3, 4]. Large numbers of inflammatory signaling enzymes such as nonreceptor protein tyrosine kinases (e.g., Src, Syk, and Janus kinase [JAK]-2), serine-threonine protein kinases (e.g., phosphoinositide 3-kinase [PI3K], phosphoinositide-dependent kinase 1 [PDK1], Akt [protein kinase B], and inhibitor of *κ*B*α* [I*κ*B*α*] kinase [IKK]) activate transcription factors such as nuclear factor (NF)-*κ*B and activator protein (AP)-1 [5, 6]. In response, numerous inflammatory genes are expressed such as pro-TNF-*α* for TNF-*α* secretion, inducible NO synthase (iNOS) for NO release, and cyclooxygenase (COX)-2 for PGE_2_ production [7–10].

**Figure 1:**
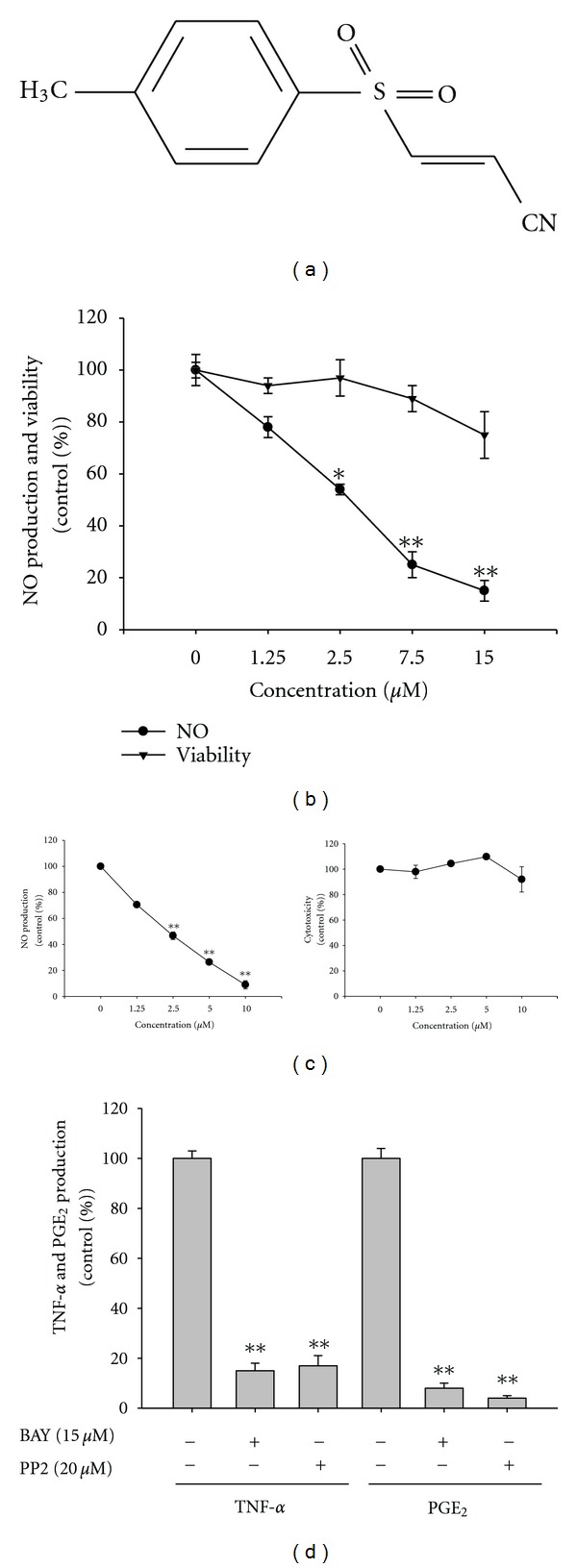
Effect of BAY (BAY 11-7082) on the production of NO, TNF-*α*, and PGE_2_ in RAW264.7 and peritoneal macrophages, and cell viability. (a) Chemical structure of BAY. (b, c left panel, and d) Levels of NO determined by the Griess assay, TNF-*α* by ELISA, and PGE2 by EIA from culture supernatants of RAW264.7 cell (b and d) and peritoneal macrophages (c) treated with BAY (0 to 15 *μ*M) in the presence or absence of LPS (1 *μ*g/mL) for 6 h (TNF-*α*) or 24 h (NO and PGE_2_). (b and c right panel) Viability of RAW264.7 cells treated with BAY by MTT assays. *P < 0.05 and **P < 0.01 compared to the control.

BAY11-7082 (BAY, Figure 1(a)) is a representative IKK inhibitor. Although IKK/NF-*κ*B is important biochemical factor, the pharmacological activities of BAY such as inhibition of inflammatory cytokines [11], induction of heme oxygenase-1 [12], suppression of ICAM-1 expression [13], reduction of ATPase activity of NLRP3 inflammasome [14], and increase in neutrophil apoptosis [15] strongly indicate that it is not a selective inhibitor of only IKK. Therefore, in this study, we explored its inhibitory potency for inflammatory signals leading to the production of NO, PGE_2_, and TNF-*α* under LPS-activated conditions, using primary and cancerous (RAW264.7 cells) macrophages.

## 2. Materials and Methods

### 2.1. Materials

BAY 11-7082, or (E)-3-(4-methylphenylsulfonyl)-2-propenenitrile; lipopolysaccharide (LPS; *Escherichia coli* 0111:B4); and 3-4,5-(dimethylthiazol-2-yl)-2,5-diphenyltetrazolium bromide (MTT) were from Sigma Chemical Co. (St. Louis, MO, USA). LY294002, wortmannin, U0126 (U0), SB203580 (SB), SP600125 (SP), and PP2 were from Calbiochem (La Jolla, CA, USA). Luciferase constructs containing promoters sensitive to NF-*κ*B, CREB, and AP-1 were gifts from Profs. Hae Young Chung (Pusan National University, Pusan, Korea) and Man Hee Rhee (Kyungpook National University, Daegu, Korea). Enzyme immunoassay (EIA) kits and enzyme-linked immunosorbent assay (ELISA) kits for determining PGE_2_ and TNF-*α* were from Amersham (Little Chalfont, Buckinghamshire, UK). Fetal bovine serum and RPMI1640 were from Gibco (Grand Island, NY, USA). The murine macrophage cell line RAW264.7 and the human embryonic kidney cell line HEK293 were from the ATCC (Rockville, MD, USA). All other chemicals were of analytical grade and from Sigma. Phosphospecific or total antibodies to p65, p50, Src, Syk, PDK1, p85, Akt, I*κ*B*α*, p38, ERK, JNK, lamin A/C, and *β* actin were from cell signaling (Beverly, MA, USA).

### 2.2. Mice

Six-week-old male C57BL/6 and ICR mice were from DAEHAN BIOLINK (Chungbuk, Korea). Mice were given food pellets (Samyang, Daejeon, Korea) and water *ad libitum* under a 12-h light/dark cycle. Studies were performed in accordance with guidelines established by the Kangwon National University Institutional Animal Care and Use Committee.

### 2.3. Preparation of Peritoneal Macrophages

Peritoneal exudates were obtained from male C57BL/6 mice (7-8 weeks old, 17–21 g) by lavaging 4 days after intraperitoneal injection of 1 mL of sterile 4% thioglycollate broth (Difco Laboratories, Detroit, MI), as reported previously [16]. After washing with RPMI 1640 medium containing 2% fetal bovine serum (FBS), peritoneal macrophages (1 × 10^6^ cells/mL) were plated in 100 mm tissue culture dishes for 4 h at 37°C in a 5% CO_2_-humidified atmosphere.

### 2.4. Cell Culture

RAW264.7 and HEK293 cells were cultured in DMEM or RPMI1640 medium supplemented with 10% heat-inactivated FBS (Gibco, Grand Island, NY, USA), glutamine, and antibiotics (penicillin and streptomycin) at 37°C under 5% CO_2_. Cells were detached with a cell scraper. At the cell density used for the experiments (2 × 10^6^ cells/mL), the proportion of dead cells was less than 1% as measured by trypan blue dye exclusion.

### 2.5. Determination of NO, PGE_2_, and TNF-*α*

After preincubation of RAW264.7 cells (1 × 10^6^ cells/mL) or bone marrow-derived macrophages (2 × 10^6^ cells/mL) for 18 h, cells were pretreated with BAY (0 to 15 *μ*M) for 30 min and further incubated with LPS (1 *μ*g/mL) for 6 (TNF-*α*) or 24 (NO and PGE_2_) h. Levels of NO, PGE_2_, and TNF-*α* were determined with Griess reagent and ELISA kits as described previously [17, 18].

### 2.6. Cell Viability Test

After preincubation of RAW264.7 or HEK293 cells (2.5 × 10^6^ cells/mL) for 18 h, BAY (0 to 15 or 30 *μ*M) was added to the cells and incubated for 2 or 24 h. The cytotoxic effect of BAY was evaluated by a conventional MTT assay, as reported previously [19, 20]. At 1 or 3 h before culture termination, 10 *μ*L MTT solution (10 mg/mL in phosphate-buffered saline, pH 7.4) was added to each well, and the cells were continuously cultured until termination of the experiment. Incubation was halted by the addition of 15% sodium dodecyl sulfate (SDS) into each well, solubilizing the formazan [21]. Absorbance at 570 nm (OD_570–630_) was measured using a SpectraMax 250 microplate reader.

### 2.7. Preparation of Cell Lysates and Nuclear Fraction, and Immunoblotting

RAW264.7 cells (5 × 10^6^ cells/mL) were washed three times in cold PBS with 1 mM sodium orthovanadate and lysed by a sonicator in lysis buffer (20 mM tris-HCl, pH 7.4, 2 mM EDTA, 2 mM ethyleneglycotetraacetic acid, 50 mM *β*-glycerophosphate, 1 mM sodium orthovanadate, 1 mM dithiothreitol, 1% Triton X-100, 10% glycerol, 10 *μ*g/mL aprotinin, 10 *μ*g/mL pepstatin, 1 mM benzimide, and 2 mM PMSF) for 30 min with rotation at 4°C. Lysates were clarified by centrifugation at 16,000 xg for 10 min at 4°C and stored at −20°C until needed.

Nuclear lysates were prepared in a three-step procedure [22]. After treatment, cells were collected with a rubber policeman, washed with PBS, and lysed in 500 *μ*L lysis buffer containing 50 mM KCl, 0.5% Nonidet P-40, 25 mM HEPES (pH 7.8), 1 mM phenylmethylsulfonyl fluoride, 10 *μ*g/mL leupeptin, 20 *μ*g/mL aprotinin, and 100 *μ*M 1,4-dithiothreitol (DTT) on ice for 4 min. Cell lysates were centrifuged at 19,326 xg for 1 min in a microcentrifuge. In the second step, the nuclear fraction pellet was washed once in washing buffer (lysis buffer without Nonidet P-40). In the final step, nuclei were treated with an extraction buffer of lysis buffer with 500 mM KCl and 10% glycerol. The nuclei/extraction buffer mixture was frozen at −80°C, thawed on ice and centrifuged at 19,326 xg for 5 min. The supernatant was collected as a nuclear extract. Soluble cell lysates were immunoblotted and protein levels determined as previously reported [23].

### 2.8. Luciferase Reporter Gene Activity Assay

HEK293 cells (1 × 10^6^ cells/mL) were transfected with 1 *μ*g of plasmid containing NF-*κ*B-Luc, IFN-*β*-promoter-Luc, CREB-Luc, or AP-1-Luc as well as *β*-galactosidase using the polyethylenimine (PEI) method in a 12-well plate according to a previous report [24]. Cells were used 48 h after transfection. Luciferase assays were performed using the Luciferase Assay System (Promega) as reported previously [25].

### 2.9. mRNA Analysis by SemiQuantitative Reverse Transcriptase- and Real-Time Polymerase Chain Reactions

To determine cytokine mRNA expression levels, total RNA was isolated from LPS-treated RAW264.7 cells with TRIzol Reagent (Gibco), according to the manufacturer's instructions. Total RNA was stored at −70°C until use. Semiquantitative reverse transcriptase (RT) reactions were conducted as reported previously [26, 27]. Quantification of mRNA was performed using real-time RT-PCR according to the manufacturer's instructions for SYBR Premix Ex Taq (Takara Bio, Inc., Shiga, Japan) using a real-time thermal cycler (Bio-Rad, Hercules, CA, USA) as reported previously [28]. Results were expressed as optimal density ratios to GAPDH. Primers (Bioneer, Daejeon, Korea) are in Table 1.

### 2.10. Statistical Analysis

**Figure 2:**
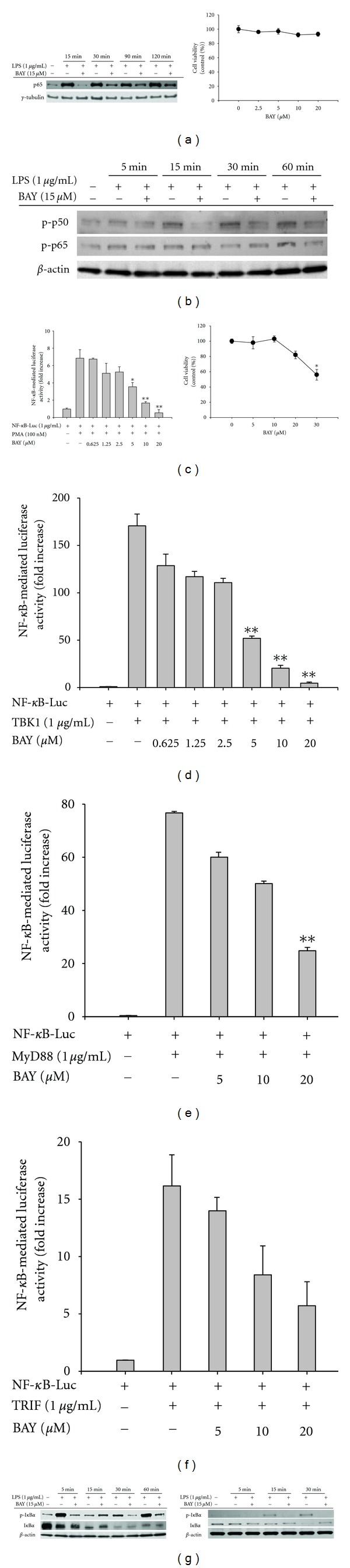
Effect of BAY on transcriptional activation of NF-*κ*B. (a left panel, b, and g) Translocated or phosphorylated levels of NF-*κ*B subunits from nucleus (a) or whole lysates (b) of RAW264.7 cells (a, b, and g left panel) or peritoneal macrophages (g right panel) treated with BAY (15 *μ*M) in the presence or absence of LPS (1 *μ*g/mL) for indicated times, evaluated by immunoblotting. (c left panel to f) HEK293 cells cotransfected with NF-*κ*B-Luc construct (1 *μ*g/mL) and *β*-gal (transfection control) were treated with BAY (0 to 20 *μ*M) in the presence or absence of PMA (100 nM) or by cotransfection with NF-*κ*B activation inducers (MyD88, TRIF, and TBK1). Luciferase activity was measured using a luminometer. (a right panel and c right panel) Viability of RAW264.7 (a right panel) and HEK293 (c right panel) cells treated with BAY (0 to 20 or 30 *μ*M) for 2 or 24 h was examined by MTT assays. *P < 0.05 and **P < 0.01 compared to the control.

Data (Figures 1(b)–1(d), 2(c)–2(f), 4(b)–4(e), 4(g), 5(a), 5(b), and 5(e)) are expressed as the mean with standard deviation (SD) as calculated from one (*n* = 6) of two independent experiments. Other data are representative of three different experiments with similar results. For statistical comparisons, results were analyzed using Mann-Whitney test. All *P*  values < 0.05 were considered statistically significant. All statistical tests were conducted using SPSS (SPSS Inc., Chicago, IL, USA).

## 3. Results and Discussion

As previously reported, BAY showed strong inhibition of NF-*κ*B activation. BAY blocked the production of NO, PGE_2_, and TNF-*α*, well-known inflammatory responses generated by activated NF-*κ*B [29], in LPS-treated RAW264.7 cells (Figures 1(b) and 1(d)) and peritoneal macrophages (Figure 1(c)). It suppressed the translocation of NF-*κ*B subunit (p65) in a time-dependent manner (Figure 2(a) left panel) and blocked the phosphorylation of p50 and p65 in whole cell lysates (Figure 2(b)), without altering cell viability up to 20 *μ*M (Figure 2(a) right panel), indicating that it could specifically block the activation and translocation pathway of NF-*κ*B as reported previously [30]. As shown in Figures 2(c)–2(f), BAY (0 to 20 *μ*M) diminished the activity of NF-*κ*B induced by PMA, a cell permeable PKC activator, and adaptor or signaling molecules of TLR (TRIF, MyD88, and TBK1), as assessed by reporter gene assay. Furthermore, I*κ*B*α* phosphorylation [31], a representative upstream pathway for NF-*κ*B translocation, was also strongly suppressed in LPS-treated RAW264.7 cells (Figure 2(g) left panel) and peritoneal macrophages (Figure 2(g) right panel), as reported previously [32], due to its direct inhibition of IKK [33].

**Figure 3:**
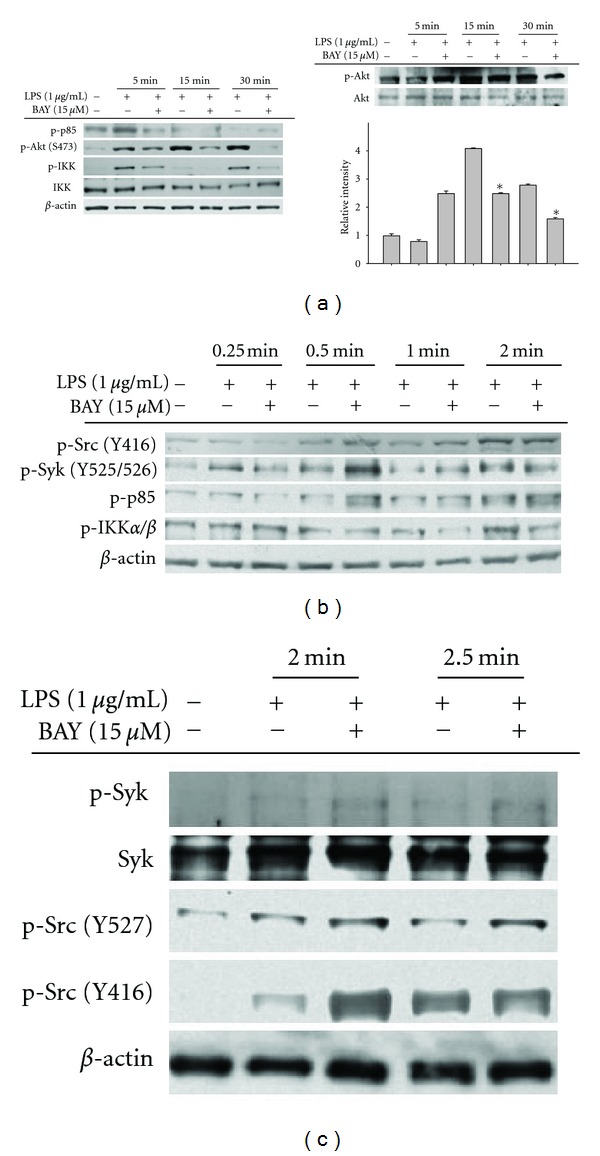
Effect of BAY on the activation of upstream signaling for NF-*κ*B translocation. (a to c) RAW264.7 cells (a left panel, b, and c) or peritoneal macrophages (a right panel) (5 × 10^6^ cells/mL) were incubated with BAY (15 *μ*M) in the presence or absence of LPS (1 *μ*g/mL) for indicated times. After preparing whole lysates, levels of phospho (p)- or total proteins of I*κ*B*α*, IKK*α*/*β*, Akt, p85/PI3K, Syk, and Src were identified by immunoprecipitation. Relative intensity (a right panel) was calculated by densitometric scanning.

Unexpectedly, however, the pharmacological activity of BAY seemed not to be limited to IKK inhibition. It clearly suppressed IKK phosphorylation (Figure 3(a) left panel), mediated by Akt in inflammatory signaling. Furthermore, it blocked the phosphorylation of Akt in both LPS-treated RAW264.7 cells (Figure 3(a) right panel) and peritoneal macrophages (Figure 3(a) right panel), indicating that the target of BAY in inflammatory signaling was upstream of IKK and Akt. Since Src and Syk are representative upstream kinases that activate PI3K, PDK1, and Akt by cascade phosphorylation [34], we evaluated whether these enzymes were suppressed by BAY. As shown in Figures 3(b) and 3(c), BAY did not suppress the phosphorylation of p85, a regulatory subunit of PI3K [35], or its upstream kinases Syk and Src, but diminished p-IKK levels at 2 min. This suggested that the target of BAY in the NF-*κ*B inhibitory pathway was not IKK, but a protein activated upstream of IKK and downstream of Src and Syk. Currently, we do not know which enzyme directly contributes to inhibition by BAY. One of three PI3K isoforms or PDK1 could be the actual target that is regulated by BAY. We plan to conduct future experiments to determine this.

**Figure 4:**
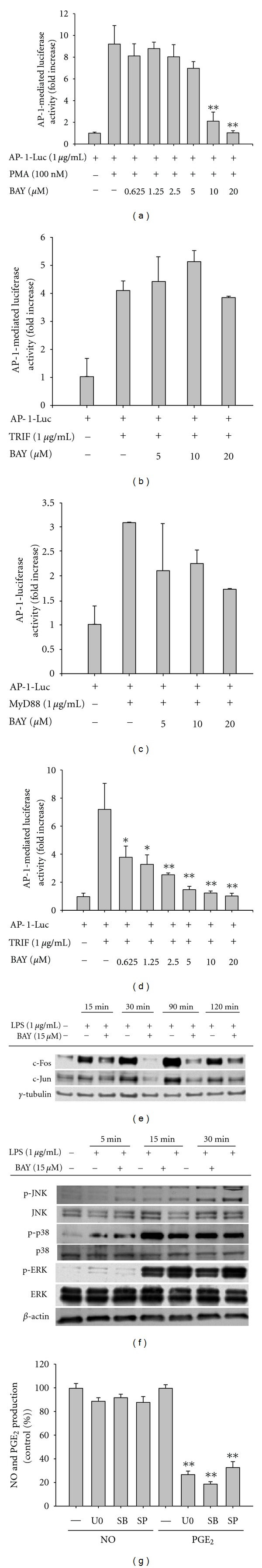
Effect of BAY on the activation of the AP-1 pathway. (a to d) HEK293 cells cotransfected with AP-1-Luc construct (1 *μ*g/mL) and *β*-gal (as a transfection control) were treated with BAY (0 to 20 *μ*M) in the presence or absence of PMA (100 nM) or by cotransfection with AP-1 activation inducers (MyD88, TRIF, and TBK1). Luciferase activity was measured using a luminometer. (e) Translocated or phosphorylated levels of AP-1 family (c-Fos and c-Jun) from the nucleus from RAW264.7 cells treated with BAY (15 *μ*M) in the presence or absence of LPS (1 *μ*g/mL), evaluated by immunoblotting. (f) RAW264.7 cells (5 × 10^6^ cells/mL) were incubated with BAY (15 *μ*M) in the presence or absence of LPS (1 *μ*g/mL) for indicated times. After preparing total lysates, levels of phospho (p)- or total proteins of JNK, p38, and ERK were identified by immunoprecipitation. (g) Levels of NO determined by the Griess assay and PGE2 by EIA from culture supernatants of RAW264.7 cell treated with U0126 (U0), SB203580 (SB), or SP600125 (SP) in the presence or absence of LPS (1 *μ*g/mL) for 24 h. *P < 0.05 and **P < 0.01 compared to the control.

In addition to NF-*κ*B, AP-1 is a representative transcription factor that is activated in inflammatory responses [36]. Therefore, we examined whether BAY blocked the activation of AP-1 in LPS-treated RAW264.7 cells, by measuring the nuclear translocated level of AP-1 components, c-Jun and c-Fos, and using a reporter gene (luciferase) assay with DNA construct containing an AP-1-binding promoter region. As seen in Figures 4(a)–4(d), BAY suppressed the promoter-binding activity of AP-1 induced by PMA and TBK1, but not TRIF and MyD88, as assessed by luciferase activity. Similarly, BAY also strongly suppressed nuclear translocation of c-Fos and c-Jun (Figure 4(e)), indicating that it could modulate the activation pathway required for the translocation of AP-1. Indeed, BAY reduced important upstream events for AP-1 translocation. Thus, it diminished the phosphorylation levels of ERK at 5 min and p38 at 15 min although it also increased the phosphorylation of ERK at 15 to 30 min (Figure 4(f)). Therefore, our data suggested that BAY had a negative effect on AP-1 activation pathway, managed by PKC and TBK1, via indirectly suppressing MAPK (ERK and p38) activation pathway. The fact that U0126 (U0), an ERK inhibitor, and SB203580 (SB), a p38 inhibitor, blocked the production of PGE_2_ and TNF-*α* (data not shown), but not NO (Figure 4(g)), implies that BAY-mediated inhibition of ERK and p38 phosphorylation did not affect its NO inhibitory action.

**Figure 5:**
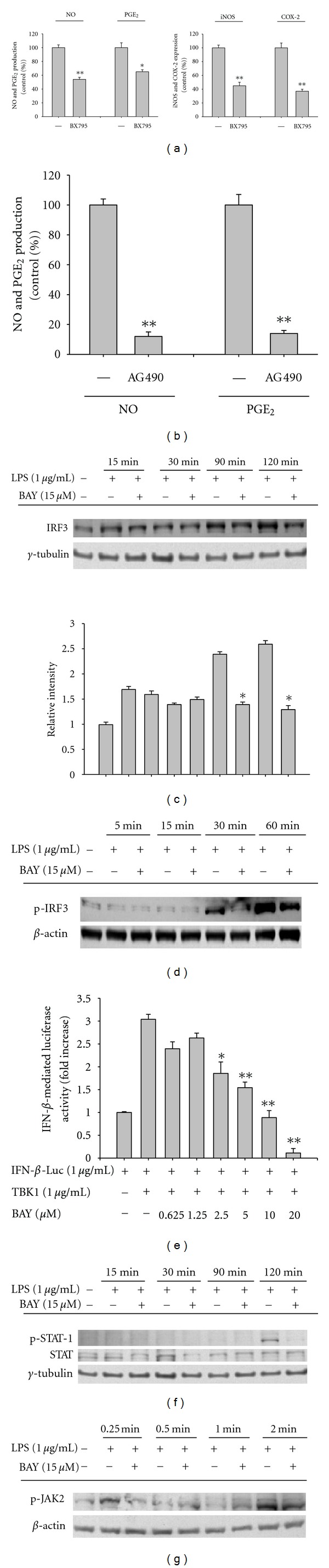
Effect of BAY on the activation of the IRF-3 pathway. (a left panel and b) Levels of NO by the Griess assay and PGE_2_ by EIA from culture supernatants of RAW264.7 cell treated with BX795 (5 *μ*M) or AG490 (20 *μ*M) in the presence or absence of LPS (1 *μ*g/mL) for 24 h. (a right panel) Levels of COX-2 and iNOS mRNA by real-time PCR using LPS-treated RAW274.7 cells pretreated with BX795 (5 *μ*M) for 6 h. (c, d, f, and g) Translocated or phosphorylated levels of IRF-3, STAT-1 or JAK2 from nucleus (c and f) or total lysates (d and g) from RAW264.7 cells treated with BAY (15 *μ*M) in the presence or absence of LPS (1 *μ*g/mL) for indicated times, evaluated by immunoblotting. (e) HEK293 cells cotransfected with IFN-*β*-promoter-1-Luc construct (1 *μ*g/mL) and *β*-gal (as a transfection control) were treated with BAY in the presence or absence of TBK1 (1 *μ*g/mL). Luciferase activity was measured using a luminometer. Relative intensity (c) was calculated by densitometric scanning. *P < 0.05 and **P < 0.01 compared to the control.

Recent work on inflammatory signaling demonstrated a critical role for IRF-3 in releasing type I interferons such as IFN-*α* and IFN-*β* [37], and additional inflammatory responses by these cytokines via activation of the JAK2/STAT-1 pathway [38]. The activation of the JAK2/STAT-1 pathway is important in the expression of iNOS and COX-2 and other proinflammatory cytokines such as IL-1*β* and TNF-*α* [39]. Blockade of the IRF-3 pathway with BX795, a TBK1 inhibitor [40], blocked the expression of iNOS and COX-2 and suppressed the production of NO and PGE_2_ (Figure 5(a)). AG490, an inhibitor of JAK2/STAT-1 pathway also elicited strong anti-inflammatory responses (Figure 5(b)). Therefore, we examined whether these pathways were also targeted by BAY. BAY strongly suppressed the translocation of IRF3 into the nucleus (Figure 5(c)) and its phosphorylation in the cytosol (Figure 5(d)). This indicated that the IRF-3 regulatory pathway was also directly modulated by BAY. In agreement, BAY diminished the upregulation of luciferase activity induced by TBK1 (Figure 5(e)), suggesting that the TBK1-mediated inflammatory pathway was a BAY target. Suppression of the JAK2/STAT-1 pathway by BAY was determined by measuring the nuclear translocation of phospho-STAT-1 and its upstream kinase. This also strongly indicated involvement in BAY-mediated inhibition. BAY strongly suppressed the phosphorylation of STAT-1 at 120 min in the nucleus (Figure 5(f)) and JAK-2 at 0.25 and 2 min in whole cell lysates (Figure 5(g)), suggesting that the JAK-2/STAT-1-mediated inflammatory responses were also targeted by BAY.

The mechanism of broad-spectrum pharmacological activity of BAY in various inflammatory signaling pathways is not clear. The main factor of this could be derived by its structural properties. Several compounds (e.g., celecoxib, phenylpropanoid derivatives) with a methylphenyl group in their backbone display anti-inflammatory activity by inhibiting various enzyme targets [41, 42]. Recently, we have also found that 8-(tosylamino)quinoline (8-TQ), with a similar structural backbone, strongly suppresses various inflammatory signaling cascades (Jung et al., submitted). Therefore, our data suggested that a structural feature of BAY contributed to its multiple pharmacological activities. Since a compound, 3-(4-(tert-octyl)phenoxy)propane-1,2-diol, with multiple inhibitory targets such as Syk, IKK, and p38, was found to display higher *in vivo* efficacy [43], it seems to be worth to further develop BAY derivatives. In view of this, we are currently collaborating with several Chemists to synthesize and develop pharmacologically stronger BAY derivatives.

In summary, BAY exhibited broad-spectrum inhibitory activity against inflammatory signaling pathways including PI3K/Akt/IKK/NF-*κ*B, ERK/JNK/AP-1, TBK1/IRF-3, and JAK-2/STAT-1. The suppressive activity of BAY was linked to the suppression of NO, TNF-*α*, and PGE_2_ release. Although the direct target of BAY needs to be identified, our data strongly implied that BAY inhibited the TLR4-activated signaling cascade and the subsequent inflammatory response by targeting multiple signaling enzymes and transcription factors. Considering that inflammatory responses occurred through multiple signaling pathways, and simultaneous inhibition of these pathways contributes to maximum therapeutic potential, the chemical optimization of BAY could be helpful in developing strong BAY-derived anti-inflammatory drugs with multiple targets.

## Figures and Tables

**Table 1:** Real-time PCR primers.

Name		Sequence (5′ to 3′)
iNOS	F	GGA GCC TTT AGA CCT CAA CAG A
	R	TGA ACG AGG AGG GTG GTG
COX-2	F	CACTACATCCTGACCCACTT
	R	ATGCTCCTGCTTGAGTATGT
GAPDH	F	CAA TGA ATA CGG CTA CAG CAA C
	R	AGG GAG ATG CTC AGT GTT GG
